# Burden, screening, and treatment of depressive and anxious symptoms among women referred to cardiac rehabilitation: a prospective study

**DOI:** 10.1186/s12905-017-0367-1

**Published:** 2017-02-07

**Authors:** Megan C. Hurley, Heather M. Arthur, Caroline Chessex, Paul Oh, Karam Turk-Adawi, Sherry L. Grace

**Affiliations:** 1 School of Kinesiology and Health Science, York University, 368 Bethune College-4700 Keele Street, Toronto, Ontario M3J 1P3 Canada; 2 Hamilton Health Sciences Centre, Hamilton, Ontario Canada; 3 University Health Network, Toronto, Ontario Canada; 4 Public Health Department, Qatar University, Doha, Qatar

**Keywords:** Cardiac rehabilitation, Women, Screening, Depression, Anxiety

## Abstract

**Background:**

Cardiovascular disease (CVD) is one of the leading causes of morbidity and mortality among women. Women with CVD experience a greater burden of psychosocial distress than men, and practice guidelines promote screening in cardiac patients, especially women. The objectives herein were to describe the burden of psychosocial distress, extent of screening, forms of treatment, and whether receipt of treatment was related to psychosocial distress symptom severity at follow-up, among women.

**Methods:**

Within a multi-center trial of women randomized to cardiac rehabilitation models, consenting participants were asked to complete surveys upon consent and 6 months later. Clinical data were extracted from charts. This study presents a secondary analysis of the surveys, including investigator-generated items assessing screening and treatment, the Beck Depression Inventory-II, the Hospital Anxiety and Depression Scale, and Patient Health Questionnaire-2.

**Results:**

Of the 128 (67.0%) participants with valid baseline and follow-up survey results, 48 (40.3%) self-reported that they recalled being screened, and of these, 10 (21.3%) recalled discussing the results with a health care professional. Fifty-six (43.8%) retained participants had elevated symptoms of psychosocial distress at baseline, of which 25 (44.6%) were receiving treatment. Regression analyses showed that treatment of psychosocial distress was not significantly associated with follow-up depressive symptoms, but was significantly associated with greater follow-up anxiety.

**Conclusions:**

Findings reiterate the great burden of psychosocial distress among women with CVD. Less than half of patients with elevated symptoms were treated, and the treatment approaches appeared to insufficiently achieve symptom relief.

## Background

Globally, cardiovascular disease (CVD) is the leading cause of morbidity and mortality in women and men, representing 30% of all deaths worldwide [1]. Depression and anxiety are two of the most frequent co-morbidities associated with CVD, increasing the overall impact of disease. Approximately 30% of patients who have been hospitalized for a myocardial infarction experience depressive symptoms, of which 15–20% suffer from major depression [2]. The prevalence of anxiety has been less-studied, but there is an understandable elevation of symptoms of anxiety following an acute CVD event [3].

Both symptoms of depression and anxiety post-myocardial infarction are associated with an increased risk of experiencing recurrent cardiac events [4]. Co-morbid depression is associated with a two-time greater risk of mortality in patients with CVD [5], and is inversely related to the adoption of secondary prevention behaviors, including smoking cessation and participation in cardiac rehabilitation (CR) [2].

The prevalence of major depression in women with CVD is twice that of men. Women also tend to experience greater anxiety after a cardiac event [6].

Cardiovascular clinical practice guidelines recommend routine screening for depression following a cardiac event [7–10]. The American Heart Association recommends screening with the Patient Health Questionnaire (PHQ)-2 [7]. The limited evidence available shows few cardiac patients are screened in the inpatient setting. A recent review identified that there has been no evaluation of whether screening in the outpatient cardiology setting is useful [11]. In addition, it has not been investigated how and whether patients are being fully informed they are being screened for depression.

Evidence-based therapies for depression and anxiety are well-established [12, 13], and have been tested in the CVD population [14–17]. There is some emerging evidence that treatment, including psychotherapy, pharmacology, and a combination of the two, not only reduce psychosocial distress (i.e. symptoms of depression and anxiety) [14–18], but also can reduce the risk of recurrent CVD events [19] and improve overall prognosis [14, 20]. Exercise as part of CR has also been shown to reduce depressive symptoms, as well as morbidity and mortality [19].

There are some reports of unsuccessful treatment of co-morbid depression among women, and in fact that psychosocial treatment may be associated with adverse outcomes. The results of the Montreal Heart Attack Readjustment Trial [21] demonstrated that women in the treatment arm displayed slightly higher cardiac and all-cause mortality compared to usual care and minimal improvements in depressive and anxious symptom severity were reported. Furthermore, although not significant, results showed that outcomes for women in the Enhancing Recovery in Coronary Heart Disease Patients trial appeared better under usual care, compared to men in whom outcomes were more favorable with treatment [15]. Results of these studies suggest that a better understanding is needed of effective psychological therapies for women with CVD [15, 21].

The objectives of this study were to: (1) describe rates of psychosocial distress screening recall, as well as outcome of such screening. In addition, (2) describe the number of women considered to be experiencing elevated symptoms of depression and/or anxiety. In women with elevated symptoms of psychosocial distress, the aims were to: (2a) describe the proportion treated, (2b) the types of treatments received, and (2c) the type of provider from whom they received treatment. In the case of pharmacological treatment, the aims were to: (2d) describe the class of medications prescribed, (2e) patient adherence, and (2f) if the medication had been changed or titrated between baseline and follow-up testing. Finally, the last objective was to: (3) describe whether receipt of treatment was related to depressive/anxious symptom severity at follow-up.

## Methods

### Design and procedure

This study presents a secondary analysis of a single-blind pragmatic randomized controlled trial of female outpatients with CVD randomized to one of three types of CR, to understand the effects of program model on adherence [22]. The design herein for this sub-study is observational and prospective. The study was approved by the Research Ethics Boards of the participating hospitals, as well as York University’s Office of Research Ethics (2009-323).

Participants were recruited from six inpatient and outpatient cardiac units in the Greater Toronto Area of Ontario, Canada. Written consent was obtained from all eligible and willing participants. Participants were then randomized to one of three CR program models: (1) mixed-sex traditional CR, (2) women-only hospital-based CR, and (3) home-based CR. Participants in the women-only model who were not participating in the larger trial were also approached to participate in an observational sub-study, where the same assessments were administered.

CR was delivered in 3 centres; 1 centre had a part-time psychologist and social worker and the others had established relationships with mental healthcare providers to whom they could refer patients external to their program. Each program and model were delivered in accordance with the Canadian CR clinical practice guidelines [10], including an initial assessment of risk factors including depression symptoms. The 3 centers routinely administer a paper-and-pencil depression survey. Given this consistency, model attended would not have an impact on the objectives herein (for example differences in screening by program model were tested and no difference was observed; *p* = .60). CR programs were 4–6 months in duration.

Clinical information was extracted from inpatient and/or outpatient medical charts. Prior to CR initiation, participants were asked to complete a self-report survey, which included a number of standardized and validated scales assessing psychosocial distress, medication adherence, medication use, exercise behavior and socio-demographic characteristics.

Follow-up assessments occurred 6 months later by mail. The self-report survey again assessed psychosocial distress, medication adherence, medication use, and exercise behavior. Moreover, an audit of CR charts was undertaken to ascertain CR enrollment and completion. Response rate was optimized through repeated and personalized contacts [23]. This included a replacement survey mailing and telephone calls, if required.

### Participants

Participants in this study were consenting women inpatients or outpatients with documented coronary artery disease, and/or acute coronary syndrome, and/or undergoing revascularization (percutaneous coronary intervention or coronary artery bypass grafting), and/or valve surgery. These chosen cardiac indications were based on accepted standard CR referral recommendations [9, 10].

Inclusion criteria were: patient resided in Toronto or Hamilton, within 45 min of CR site; proficiency in the English language, and eligibility for home-based CR. Exclusion criteria were: (1) musculoskeletal, neuromuscular, visual, cognitive or non-dysphoric serious psychiatric condition (e.g., schizophrenia), or any serious or terminal illness not otherwise specified which would preclude CR eligibility based on CR guidelines [10], (2) physician deemed patient not suitable for CR at time of intake exercise stress test, (3) patient planned to leave the region prior to the anticipated end of participation, (4) patient discharged to a long-term care facility, and (5) participation in another clinical trial with behavioral interventions.

### Measures

Socio-demographic characteristics were assessed in the initial participant survey through forced-choice response options, including: age, ethnic background, marital status, education, income, work status and dependents. Clinical data extracted from medical charts included cardiac diagnoses, risk factors, and co-morbid conditions.

Exercise behavior was also measured in the final survey, considering its relation to mood [24, 25] using the Godin Leisure-time Exercise Questionnaire [26]. This is a brief and reliable instrument that was used to assess usual leisure-time physical activity behavior during a 1-week period. Lastly, CR participation (also involving exercise) was verified via an audit of participants CR charts.

### Assessment of psychosocial distress: depressive and anxious symptoms

Psychometrically-validated scales were administered in both the baseline and follow-up self-report surveys to assess psychosocial distress. First, the Beck Depression Inventory-II (BDI-II) [27] was administered to assess depressive symptoms in the baseline survey. It is a reliable and well-validated 21-item scale that uses a 4-choice response format. It has been widely used in the general population and in populations with long-term illness, including cardiac disease. This scale has high internal consistency and good sensitivity for detection of depression in cardiac patients [28]. Higher scores reflect greater depressive symptomatology, with scores ≥14 reflecting “elevated” (i.e., mild to severe) symptomatology.

Second, the Patient Health Questionnaire-2 (PHQ-2) [29] was administered in both the baseline and follow-up surveys to assess the frequency of the two cardinal features of depression, namely depressed mood and anhedonia. The PHQ-2 total score ranges from 0 to 6, with a score of ≥3 indicating elevated symptoms [29]. This scale has been widely used in the cardiac population.

Finally, The Hospital Anxiety Depression Scale (HADS) [30] was administered in the follow-up survey, to additionally assess anxiety. The HADS is a 14-item questionnaire with seven items assessing anxiety and seven items assessing depressive symptoms (primary outcome). Each item is scored from 0 to 3, with each subscale scored out of 21. Scores of ≥9 represent elevated symptoms of anxiety or depression [30, 31]. This scale has been tested in the cardiac population, and has high internal consistency and good sensitivity [28].

### Assessment of psychosocial distress: screening recall and treatment

Investigator-generated items assessing problems with, and treatment for depression and anxiety were incorporated in both surveys. In the initial survey, participants were asked if they had current problems with depression and/or anxiety (yes/no). In both initial and final surveys, participants were also asked if they were receiving treatment (yes/no), the type of provider from whom they were receiving treatment, and finally the type of treatment they were receiving (i.e., pharmacotherapy, psychotherapy, or both). In the final survey, participants were asked whether they had been screened for depression and/or anxiety since they were referred to CR (yes/no), and the outcome of such screening.

Use of psychoactive medication was ascertained by reviewing self-reported medication lists. Participants were asked in both initial and final surveys to record all medications they were currently taking, and the dose per day. Psychoactive drugs were coded according to their drug class [32]. Whether the psychoactive medication(s) listed were different on the baseline and follow-up survey was also assessed, and where the same medication was reported at both assessment points, whether the dosage had been changed.

Finally, adherence to medication was assessed via Morisky’s Medication Adherence Scale (MMAS-4) [33], which was administered in both baseline and follow-up surveys. It is a brief and reliable instrument used to assess medication adherence. The MMAS-4 total score ranges from 0 to 4, with any score less than 2 indicating non-adherence.

### Analysis

All statistical analyses were performed using SPSS version 20 [34]. Comparisons of socio-demographic, clinical and psychosocial characteristics between participants retained and those lost-to-follow-up, as well as group differences (i.e., self-reported distress and/or elevated scores vs. no self-reported distress and no elevated scores) were performed. Scores were compared using t-tests or chi-squared tests, as appropriate, with a significance cut-off value of *p* < 0.05. The relationship between baseline and follow-up psychological distress was also explored.

To test the first objective, a descriptive analysis of the investigator-generated item in the final survey, which assessed self-reported screening of depression and anxiety and the outcome of such screening, was performed.

To test the second objective, participants who self-reported currently experiencing psychosocial distress and/or scored above the thresholds indicating elevated distress on the BDI-II or PHQ-2 were described and selected. A descriptive analysis of psychosocial treatment at baseline and at follow-up was performed. Among those receiving treatment, description of treatment and provider type was performed. To test objectives d-f, participants who were prescribed pharmacological therapy were selected. Next, a descriptive analysis of the drug classification, medication adherence, and the frequency of medication and/or dosage change from baseline to follow-up assessment was performed.

To test the final objective, a multiple linear regression was undertaken to examine whether receipt of any treatment recorded on the follow-up survey (yes/no) was associated with depressive and anxious symptom severity at the follow-up visit, in those with elevated symptoms of psychosocial distress at baseline. The model adjusted for significant group differences (i.e., self-reported distress and/or elevated scores vs. no self-reported distress and no elevated scores) in socio-demographic, clinical and psychosocial characteristics, as well as sociodemographic and clinical variables shown to be associated with follow-up depressive and anxious symptom severity at the bivariate level.

## Results

### Respondent characteristics

**Fig. 1:**
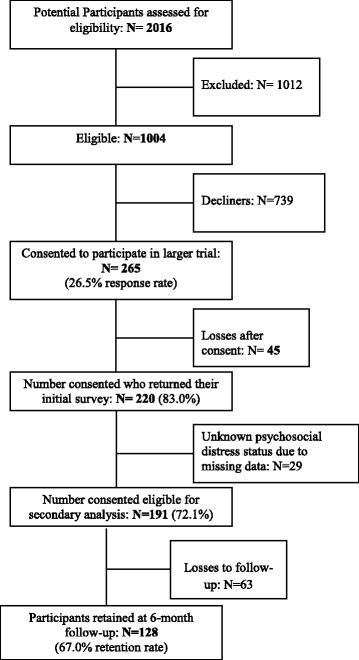
Flow Diagram

Of the 191 participants, 128 (67.0%) completed baseline and 6-month follow-up surveys analyzed in this secondary analysis. As shown in Fig. 1, 63 participants were lost to follow-up. Table 1 displays the baseline socio-demographic, clinical and psychosocial characteristics of the retained and non-retained sample. As shown, there were no significant differences in socio-demographic or clinical characteristics between groups. However, participants lost-to-follow-up (51.7%) were more likely to have a self-reported history of depression or anxiety than retained participants (32.8%). No other psychosocial differences were found between retained participants and those lost to follow-up.

**Table 1:** Participant characteristics at baseline (*N* = 191)

Characteristics	Retained participants			Lost-to follow-up	Total
	Self-reported distress and/or elevated scores *n* = 56 (43.8%)	No self- reported distress and/or elevated scores *n* = 72 (56.3%)	Total *N* = 128	*n* = 63	*N* = 191
Sociodemographic					
Age, years (mean ± SD)	63.25 ± 9.05	67.48 ± 10.81	65.63 ± 10.25^*^	64.72 ± 9.88	65.32 ± 10.11
Marital Status, n (% married)	27 (48.2)	39 (54.2)	66 (51.6)	31 (49.2)	97 (50.8)
Work Status, n (% retired)	26 (46.4)	41 (56.9)	67 (52.3)	29 (46.0)	76 (54.7)
Highest Education, n (% post-secondary)	17 (30.4)	29 (40.3)	46 (35.9)	20 (31.7)	66 (34.6)
Ethnicity, n (% white)	37 (66.1)	49 (68.1)	86 (67.2)	43 (68.3)	129 (67.5)
Annual Family Income, n (% $50000CDN or greater)	29 (51.8)	46 (63.9)	75 (58.6)	38 (60.3)	113 (59.2)
Children, n (% yes)	43 (79.6)	59 (83.1)	102 (81.6)	53 (85.5)	155 (82.9)
Clinical					
Cardiac Indication					
*PCI (% yes)*	26 (50.0)	30 (44.8)	56 (47.9)	22 (36.7)	78 (43.6)
*Angina/ACS/CAD (% yes)*	22 (43.1)	21 (31.8)	43 (36.8)	28 (46.7)	71 (40.1)
*MI (% yes)*	19 (36.5)	22 (33.3)	41 (34.7)	17 (28.3)	58 (32.6)
*CABG (% yes)*	7 (13.5)	22 (32.8)	29 (24.4)^*^	16 (26.7)	45 (25.1)
*Valve (% yes)*	9 (17.3)	17 (25.8)	26 (22.0)	9 (15.0)	35 (19.7)
BMI (mean ± SD)	30.17 ± 7.05	27.34 ± 5.52	28.91 ± 6.53^*^	29.64 ± 7.24	29.15 ± 6.75
Diabetes, n (% yes)	14 (35.0)	9 (19.1)	23 (26.4)	17 (35.4)	40 (29.6)
Hypertension, n (% yes)	32 (71.1)	44 (74.6)	76 (73.1)	39 (73.6)	115 (73.2)
Dyslipidemia, n (%)	36 (92.3)	44 (81.5)	80 (86.0)	41 (82.0)	121 (84.6)
Exercise Behavior (mean ± SD)	21.33 ± 19.74	20.86 ± 14.99	21.07 ± 17.20	16.92 ± 31.35	19.68 ± 22.93
Psychosocial					
Self-Reported History of Depression or Anxiety, n (% yes)	25 (45.5)	15 (22.4)	40 (32.8)^**^	31 (51.7)	71 (39.0)^†^
Self-reported History of Pharmacological Treatment for depression or anxiety, n (% yes)	15 (27.3)	12 (17.6)	27 (22.0)^***^	13 (22.0)	40 (22.0)
Self-reported History of Psychotherapy for depression or anxiety, n (% yes)	17 (31.5)	14 (20.9)	31 (25.6)^*^	21 (35.6)	52 (28.9)
BDI-II (mean ± SD)	16.94 ± 11.30	5.08 ± 3.59	9.89 ± 9.64^***^	11.31 ± 8.42	10.35 ± 9.26
PHQ-2 (mean ± SD)	1.90 ± 1.89	0.33 ± 0.59	1.03 ± 1.54^***^	1.43 ± 2.14	1.15 ± 1.75

*PCI* percutaneous coronary intervention, *ACS* acute coronary syndrome, *CAD* coronary artery disease, *CDN* Canadian dollars, *MI* myocardial infarction, *CABG* coronary artery bypass graft, *BMI* body mass index, *BDI* beck depression inventory, *PHQ* patient health questionnaire

^*^Denotes difference between participants with elevated vs. no elevated symptoms (^*^ *p* <0 .05; ^**^ *p* <0 .01; ^***^ *p* < 0.001)

^†^Denotes difference between participants who were retained vs. not retained at 6 month follow-up (^†^ *p* < 0.05; ^†^ *p* < 0.01; ^†††^ *p* <0.001)

### Burden of psychosocial distress

As also shown in Table 1, there were 56 (43.8%) retained participants who were experiencing elevated symptoms of psychosocial distress at baseline. Those with elevated distress symptoms were significantly more likely to have a self-reported history of depression or anxiety, as well as self-reported history of treatment for depression or anxiety than those without distress. Participants with elevated symptoms were also significantly younger, had a higher body mass index, and were more likely to have undergone percutaneous coronary intervention (PCI) than participants without elevated symptoms.

At follow-up, 14 (10.8%) had elevated scores on the HADS-D and 37 (28.7%) on the HADS-A. Based on the PHQ-2, 44 (35.8%) participants were considered to be experiencing elevated symptoms of psychosocial distress (no significant difference between the number of women with elevated symptoms at baseline and follow-up; McNemar test *p* > 0.05). A significant reduction between baseline and follow-up PHQ-2 symptom scores was found for women with elevated symptoms at baseline (mean difference =−0.62; paired t = 2.96, *p* < 0.01). Table 2 displays follow-up psychosocial indicators among women with elevated symptoms of psychosocial distress and those without at baseline. At follow-up, those with elevated symptoms at baseline still had significantly higher symptom scores on psychometrically-validated scales than participants without elevated symptoms, but their mean scores fell below the established cut-offs. Those with elevated symptoms at baseline were also more likely to report psychosocial distress screening at follow-up than those without elevated symptoms. No differences in exercise behavior, medication adherence or CR use were observed.

**Table 2:** Follow-up psychosocial and other indicators among women by psychosocial distress status at baseline (*N* = 128)

Variable	Self-reported distress and/or elevated scores *n* = 56 (43.8%)	No self-reported distress and/or elevated scores *n* = 72 (56.3%)	Total
Psychosocial Symptoms^a^			
*Depressive Symptoms (PHQ-2)*	1.37 ± 1.67	0.42 ± 0.95	0.83 ± 1.39^***^
*Depressive Symptoms (HADS-D)*	5.16 ± 4.08	2.14 ± 2.11	3.45. ± 3.45^***^
*Anxious Symptoms (HADS-A)*	7.96 ± 4.55	3.57 ± 2.99	5.47 ± 4.32^***^
Psychosocial Distress Screening (% yes)	25 (47.2)	17 (24.6)	42 (34.4)^**^
Exercise Behaviour (Godin)^a^	29.19 ± 27.51	34.99 ± 33.14	32.43 ± 30.79
Medication Adherence (MMAS)^a^	3.50 ± 0.81	3.64 ± 0.68	3.58 ± 0.74
Enrolled in CR (% yes)	48 (85.7)	65 (91.5)	113 (89.0)
Completed CR (% yes)	34 (77.3)	46 (88.5)	80 (83.3)

*PHQ* patient health questionnaire, *HAD-A and HAD-D* hospital anxiety and depression scale, *MMAS* Morisky’s medication adherence scale

^*^ *p* < 0.05; ^**^ *p* <0 .01; ^***^ *p* < 0.001 for t-test or chi-square comparing indicators by psychosocial distress status, as appropriate

^a^mean and standard deviation

### Screening

Forty-two (34.4%) participants self-reported on the follow-up survey that they recalled being asked about their mood and/or anxiety by any healthcare provider during the course of their medical care. Forty-eight (40.3%) participants reported they recalled being formally screened for psychosocial distress, of which 33 (27.7%) reported completing a paper-and-pencil screen, 6 (5.0%) a structured interview, and 9 (7.6%) reported completing both. At baseline, 18 (15.3%) participants scored above the American Heart Association’s screening threshold on the PHQ-2, at which point follow-up administration of the PHQ-9 is recommended^7^. Finally, the number of participants who reported being asked about their mood and undergoing formal screening did not significantly differ in those who ultimately enrolled in CR following referral versus those who did not (*p* = 0.46 and *p* = 0.11, respectively).

Of those who reported being formally screened, 10 (21.3%) had their results discussed with them. The outcomes of formal screening were (participants were asked to check all that apply): appropriately nothing (*n* = 16, 42.1%), pharmacology prescription (*n* = 6; 15.8%), referral to a mental health professional (*n* = 5; 10.4%), follow-up by a healthcare provider (*n* = 6; 15.8%), current problems with mood or anxiety and nothing (*n* = 4; 10.5%), referral for other mental health treatment (*n* = 1; 2.7%), other (*n* = 5; 12.8%), and unknown by participant (*n* = 2; 5.1%).

With regard to the relationship between screening and psychosocial distress, there were no significant differences in depressive symptom severity at baseline (BDI-II: 12.27 ± 12.25 vs. 8.33 ± 7.57; *p* = 0.09) or at follow-up (HADS-D: 3.83 ± 4.00 vs. 3.13 ± 3.09; *p* = 0.28) (HADS-A: 6.25 ± 4.30 vs. 4.89 ± 4.41; *p* = 0.10), between participants who recalled being screened and those who did not. With regard to the PHQ-2, there was no significant difference in scores at baseline (1.35 ± 1.62 vs. 0.85 ± 1.50; *p* = 0.10) between participants who recalled screening and those who did not, but there was a significant difference found at follow-up (1.21 ± 1.80 vs. 0.57 ± 1.00; *p* = 0.03) with those who recalled screening scoring higher.

### Treatment of distressed women

Ninety-two (48.2%) women were considered to be experiencing elevated psychosocial distress at the initial assessment (of which 56 participants were retained at follow-up). Of these 56, 25 (44.6%) reported receiving some form of treatment at baseline. Of these, 20 (80.0%) reported still receiving treatment at follow-up, and 5 (20.0%) reported they were not. Again, of those 56 distressed women at baseline, 26 (48.1%) were receiving some form of treatment at follow-up (i.e., 20 participants + 6 new). The number of participants receiving treatment did not significantly differ between baseline and follow-up assessment (McNemar test *p* > 0.05). Table 3 displays the types of treatment, as well as who was providing or supervising it, at both assessment points. As shown, most participants received medication, followed by one-fourth receiving a combination of medication and counselling, with the least proportion of treated participants receiving counselling alone.

**Table 3:** Treatment of patients who were considered distressed at baseline, by assessment point

	Baseline (*n* = 25; 44.6% treated)	Follow-up (*n* = 26; 48.1% treated)
Treatment Type		
*Medication*	15 (60.0)	17 (65.4)
*Combination*	6 (24.0)	7 (26.9)
*Counseling*	4 (16.0)	2 (7.7)
Type of Provider^a^		
*Family Doctor*	16 (69.6)	19 (82.6)
*Psychiatrist or Psychologist*	7 (30.4)	7 (30.4)
*Nurse*	1 (4.3)	2 (9.1)
*Cardiologist*	1 (4.3)	2 (8.7)
*Other*	2 (8.7)	4 (17.4)
Class of Psychoactive Medication^ab^		
*SSRI*	4 (25.0)	5 (23.8)
*Atypical antidepressant* ^*c*^	7 (43.8)	6 (28.6)
*Benzodiazepines*	4 (25.0)	8 (38.1)
*SNRI*	5 (31.3)	5 (23.8)
*TCA*	2 (12.5)	2 (10.0)

^a^Note that some women reported receiving treatment from more than one type of provider and taking more than one psychoactive medication

*SSRIs* selective serotonin reuptake inhibitors, *SNRIs* serotonin norepinephrine reuptake inhibitors, *TCA* tricyclic antidepressants

^b^Includes those treated with medication (*n* = 21 and *n* = 24, respectively)

^c^for example: Mirtazapine, Bupropion and Trazodone

Of those receiving treatment, 21 (84.0%) were taking psychoactive medications at baseline and 24 (92.3%) were taking them at follow-up. Their overall mean medication adherence score was 3.60 ± 0.68 at baseline and 3.42 ± 0.97 at follow-up, with no participants considered “non-adherent at baseline and 2 (8.3%) considered “non-adherent” at follow-up. Table 3 also displays the class of psychoactive medication taken at both time points. The same medication was reported at both assessment points for 11 (84.6%) participants, for which 2 (15.4%) participants reported a change in dosage over time (one increased and one decreased). Two (15.4%) participants reported a change in psychoactive medication from baseline to follow-up: one from selective serotonin reuptake inhibitor to a serotonin norepinephrine reuptake inhibitor, and one switched medications within the atypical class.

### Treatment effect

Participants who were receiving any form of treatment at baseline had significantly higher anxious (10.12 ± 4.61 vs. 5.89 ± 3.68) symptom HADS scores at follow-up than those who were not receiving treatment (*p* < 0.001). There were no differences in depressive (6.28 ± 4.38 vs. 4.23 ± 3.61; *p* > 0.05) symptom HADS scores. Participants who were receiving any form of treatment at follow-up had significantly higher depressive (6.46 ± 4.37 vs. 3.93 ± 3.52) and anxious (10.23 ± 4.63 vs. 5.75 ± 3.34) symptom HADS scores at follow-up than those who were not receiving treatment (*p* < 0.05 and *p* < 0.001, respectively).

An analysis of treatment effects on change in depressive symptoms in distressed patients was undertaken. Among participants who were distressed at baseline, participants who received any form of treatment experienced a non-significant reduction in symptoms (2.37 ± 2.03 at pre-test and 2.00 ± 1.93 at post-test, *p* = 0.27) on the PHQ-2, whereas distressed participants not receiving treatment experienced a significant symptom reduction (1.48 ± 1.73 at pre-test and 0.60 ± 1.08 at post-test, *p* < 0.01). Table 4 displays analysis of correlates of symptoms of depression and anxiety at follow-up. Unadjusted analyses showed that greater baseline psychosocial distress symptom scores were significantly associated with greater symptom scores of depression and anxiety at follow-up. Moreover, older age was found to be significantly related to lower follow-up anxious symptom severity.

**Table 4:** Sociodemographic and clinical correlates of depressive and anxious symptom severity at follow-up (*N* = 56)

	Depression^a^ t / r	Anxiety^a^ t / r
Sociodemographic		
Age	−.174	−.277^*^
Marital Status (married)	.137	.646
Work Status (retired)	.049	−.832
Highest Education (post-secondary)	−.150	.648
Ethnicity (white)	.560	−.166
Annual Family Income ($50000CDN or greater)	−.553	−1.626
Children (yes)	−.123	−1.163
Clinical		
Cardiac Indication		
*PCI (yes)*	−1.249	−.885
*Angina/ACS/CAD (yes)*	−.998	−1.412
*MI (yes)*	−.618	−.555
*CABG (yes)*	.575	.644
*Valve (yes)*	−.741	−.517
BMI	.101	.023
Exercise Behavior (Godin)^a^	.054	−.143
Diabetes (yes)	1.502	.006
Hypertension (yes)	.326	−.800
Dyslipidemia (yes)	−.213	−1.971
CR Enrollment (yes)^a^	−.064	−.694
CR Completion (yes)^a^	−.994	−.747
Psychosocial		
Treatment (yes)^a^	2.356^*^	4.099^***^
BDI-II	.566^***^	.465^**^
PHQ-2	.573^***^	.484^***^
PHQ-2^a^	.747^***^	.735^***^

*PCI* percutaneous coronary intervention, *ACS* acute coronary syndrome, *CAD* coronary artery disease, *CDN* Canadian dollars, *MI* myocardial infarction, *CABG* coronary artery bypass graft, *BMI* body mass index, *BDI* beck depression inventory, *PHQ* patient health questionnaire

^*^ *p* < 0.05; ^**^ *p* <0 .01; ^***^ *p* < 0.001 for t-test and Pearson’s correlation (r), as applicable

^a^Assessed at follow-up. All other variables assessed at baseline

Two multiple linear regressions were performed to ascertain whether receipt of any treatment (independent variable) was related to psychosocial distress scores at follow-up (i.e., HADS-D and HADS-A; dependent variables). The model was adjusted for baseline symptom scores and age (i.e., significant differences identified in Table 4), as well as cardiac indication and self-reported history of psychosocial distress (i.e., primary significant differences identified in Table 1). PHQ-2 scores were not included due to their correlation with BDI-II scores. The results are shown in Table 5. The models overall were significant (*F* = 3.45, *p* < 0.05; *F* = 3.10, *p* = <0.05, respectively), and were amply powered (0.94 and 0.90, respectively). The effect of treatment on depressive symptoms at follow-up did not sustain adjustment, suggesting that pharmacologic or counseling treatments did not affect depressive symptom scores. The effect of treatment on anxious symptom severity did sustain adjustment, suggesting that pharmacologic or counseling treatments were related to greater anxious symptoms. As shown, depressive symptom scores at follow-up were significantly related to psychosocial distress symptom scores at baseline, but anxious symptom scores were not (trend). No other variables in the model were found to be significantly related.

**Table 5:** Adjusted multiple linear regression model examining the association of treatment with depressive and anxious symptom severity at follow-up (*N* = 56)

	Depressive symptoms					Anxiety symptoms				
Variable	β	SE	p	95% CI		β	SE	p	95% CI	
				Lower bound	Upper bound				Lower bound	Upper bound
Treatment	−.636	1.491	.673	−3.677	2.404	−3.408	1.584	.039	−6.638	−.178
Psychosocial Distress at Baseline (BDI-II)	.208	.064	.003	.077	.339	.137	.060	.053	−.002	.276
History of Psychosocial Distress	−.571	1.497	.705	−3.625	2.482	−.978	1.590	.543	−2.266	4.221
Age	−.076	.077	.331	−.234	.081	−.111	.082	.185	−.279	.056
CABG	−.300	1.849	.872	−4.070	3.470	.168	1.964	.932	−3.837	4.173

*CABG* coronary artery bypass graft, *BDI* beck depression inventory, *CI* confidence interval, *SE* standard error

## Discussion

These findings reiterate the great burden of psychosocial distress among women with CVD [35], with approximately half of women displaying elevated symptoms. Despite screening recommendations, less than half of women cardiac patients recalled being formally screened. Moreover, less than half of women with elevated symptoms were receiving treatment. Regardless, receiving treatment had no significant impact on depressive symptoms and was related to greater, not lesser anxious symptom severity. The direction of effect for this association is likely that women experiencing anxiety are more actively soliciting treatment (i.e., reverse causality). While a structured clinical interview is needed to confirm a psychological disorder and whether treatment is warranted, these findings certainly corroborate previous reports of insufficient [36, 37] and even inappropriate [38, 39] (i.e., Tricyclic Antidepressants) treatment of these important co-morbidities.

### Implications related to screening

Despite recommendations, depression is not routinely identified in cardiac patients [40], a finding that was reiterated in the current study. Shanmugasegaram et al. [35]. found that only 28.7% of coronary artery disease patients recalled being screened for depression following a cardiac hospitalization, and 32.5% of patients enrolled in CR recalled being screened. The rates in our study were somewhat higher. First, this could be explained by recent uptake of depression screening recommendations. Second, patients in this study would have had the opportunity to be screened in-hospital at the time of cardiac event, but also potentially at CR given there are screening recommendations for both settings. Third, because the sample was comprised solely of women who are known to suffer from greater rates of depression, healthcare providers may have screened more often. While the rates were closer to 50%, and patients may have forgotten they were screened, clearly the recommendations for screening have still not been consistently implemented.

The reasons for low depression screening rates likely include the critique of the American Heart Association recommendations by Thombs et al. [41]. While the results of this secondary analysis cannot be considered conclusive evidence, findings would suggest that screening recall was not related to lower depressive symptoms among female cardiac patients 6 months later. Thus, these findings suggest research is needed on actual screening in the CR setting and the effects on downstream symptoms and health outcomes [42].

There was certainly a trend towards more severe depressive symptoms at baseline in women who recalled being screened, as well as more severe symptoms of depression and anxiety at follow-up, and this may be indicative that providers are selectively screening patients they suspect may be experiencing depression (or it may reflect that women with greater distress are more likely to recall being screened). It has been suggested that case-finding for depression might be a more cost and resource-effective strategy than universal screening [41].

The CR context may present an important opportunity to screen and address comorbid depression and psychosocial distress [19]. There are CR-specific screening recommendations in Canada where the study was conducted [10], and in the United States [9]. In both countries depression screening is considered a quality indicator, although anxiety screening is not [43, 44]. Thus, it was surprising that patients who enrolled in CR following referral did not recall greater rates of depression screening than patients who did not enroll. It is hoped results from this study will initiate discussion on the role of screening in the CR setting.

### Implications for treatment

A number of studies have examined the effect of in-hospital depression screening on patient outcomes [45–47]. Results of these studies suggest that when screening is combined with “collaborative care”, patients can achieve significant reductions in depressive symptoms [45–47]. Collaborative care involves several healthcare providers working together to deliver care [48], including frequent check-ins, medication adjustments, promotion of treatment adherence and disease-related education [45, 46]. Our results suggest that few women recall being screened, and less than half of the women who were distressed received any form of treatment, so it is most unlikely that the above benefits could be achieved. It is postulated that there is insufficient capacity to ensure effective treatment is available for all patients who screen positive, given the chronic under-funding of CR services, which may serve as a deterrent to screening. Perhaps it is time to increase the availability of psychologists and psychiatrists in CR to ensure that proven models of collaborative care are instituted.

Our findings are not consistent with many of the major CVD and psychosocial distress trials, which showed that psychoactive drugs, psychotherapy, or a combination of the two, improve psychological outcomes [14–17]. Our data are pragmatic and observational, and suggest that distressed women are: (a) not getting sufficient therapy, (b) may need to change or introduce additional therapeutic modalities to achieve symptom control and remission, or (c) not adhering to the medication for a sufficient duration to achieve remission, although it is important to note that our results are based upon self-report data in only a small sample of women. In the case of pharmacological treatment, which was the primary form of treatment in the current sample, it is known that individuals being treated with psychoactive drugs are not put on an full therapeutic dose right away to reduce the chance of side effects, such that optimal treatment efficacy may not be reached without up-titration [12]. Patients should be followed-up with by their physician to observe how they are tolerating the medication, whether they are experiencing side effects, and to up-titrate the dose if needed (to balance efficacy and safety) [12]. Our results suggest patients may not be receiving adequate follow-up, as only 1/3 of those treated pharmacologically reported any change in medication (i.e., type or dose change; although we do not have data on the frequency of follow-up visits to the treating healthcare professional). It has been reported that only 20–30% of patients undergoing depression treatment receive adequate care and follow-up in the primary care setting [7].

Caution is warranted when interpreting these findings, particularly due to measurement issues. First, the protocol did not include a structured clinical interview to formally diagnose depression or anxiety, so the number of participants with a major depressive disorder or anxiety disorder is unknown, as is the appropriateness of the rates of treatment. Self-report of symptoms of depression and anxiety is subject to under-reporting bias. Moreover, it has been found that brief depression screening tools can have unacceptably low specificity rates. The PHQ-2 screening tool, which was one of measures used in this study, may have only 52% specificity in cardiac samples [49]. This suggests that approximately half of the patients who scored above the cutoff on this tool may not warrant depression treatment. Second, while the PHQ-2 was administered in both assessments, different scales were used to measure symptoms of psychosocial distress pre and post-CR (i.e., BDI-II at pre-test and HADS at post-test) due to a change made for the larger trial. The change was made by the investigators to add anxiety assessment, while limiting an increase in survey response burden which would occur by the addition of another scale. Additionally, rates of distress screening and identification may be under-reported due to possible recall bias [50, 51]. Third, screening was only ascertained through participant report, and patients may have been unaware or forgotten screening that had occurred months prior leading to under-reporting. Future research is needed where screening is verified in medical charts. Fourth, the CR programs where participants were referred in this study routinely administer a paper-and-pencil depression survey; however we did not verify this in CR charts. Taken together, the incorporation of longitudinal data and medical record information in combination with our prospective self-report data would have enabled more fulsome testing of some of our objectives.

Fifth, due to the nature of the study design, causality in the relationship between the variables of interest and symptom control cannot be inferred. Sixth, results are limited by selection and retention bias. With regard to the former, it is not known how participants who consented to participate in the randomized trial from which this sample was drawn differed from those who did not, however previous research has suggested that there are some important differences which may impact the generalizability of these findings [52]. With regard to the latter, while the only observed difference between those retained and lost to follow-up was in the rate of self-reported history of depression or anxiety, there may be unmeasured factors which introduce a bias in our retained sample. Seventh, because the associations assessed herein were not tested on a primary basis, the required sample size for sufficient power was not determined a priori. Post-hoc calculations revealed the comparison of depressive symptom scores at post-test by receipt of treatment was well-powered (.98), however the comparison by screening recall was not (.46). Replication is warranted to test the latter association, in an appropriately-powered sample. Finally, we did not assess the history of psychosocial distress in the patients, when they were diagnosed and how this may have inter-related with their development of CVD.

## Conclusion

These findings reiterate the great burden of psychosocial distress in women cardiac outpatients. Despite guideline recommendations, screening recall was low and unrelated to depressive symptom severity 6 months later. Where treated, most women were often prescribed psychoactive medication under the supervision of their family physicians. However, symptom reduction in those receiving any form of treatment was not achieved. More research investigating the effects of screening and developing cost-effective mental health interventions for cardiac patients in the CR setting is needed.

## Abbreviations

ACS: Acute coronary syndrome
BDI: Beck depression inventory
BMI: Body mass index
CABG: Coronary artery bypass graft
CAD: Coronary artery disease
CDN: Canadian dollars
CI: Confidence interval
CR: Cardiac rehabilitation
CVD: Cardiovascular disease
HADS: Hospital anxiety and depression scale
MI: Myocardial infarction
MMAS: Morisky’s medication adherence scale
PCI: Percutaneous coronary intervention
PHQ: Patient health questionnaire
SE: Standard error
SNRIs: Serotonin Norepinephrine reuptake inhibitors
TCA: Tricyclic antidepressants
